# Agreement of measured and calculated serum osmolality during the infusion of mannitol or hypertonic saline in patients after craniotomy: a prospective, double-blinded, randomised controlled trial

**DOI:** 10.1186/s12871-015-0119-4

**Published:** 2015-10-07

**Authors:** Qian Li, Han Chen, Jing-Jing Hao, Ning-Ning Yin, Ming Xu, Jian-Xin Zhou

**Affiliations:** Department of Critical Care Medicine, Beijing Tiantan Hospital, Capital Medical University, Beijing, China

**Keywords:** Serum osmolality, Measurement, Estimation, Correlation, Mannitol, Hypertonic saline, Craniotomy

## Abstract

**Background:**

Mannitol and hypertonic saline are used to ameliorate brain edema and intracranial hypertension during and after craniotomy. We hypothesized that the agreement of measured and calculated serum osmolality during the infusion of hypertonic saline would be better than mannitol. The objective was to determine the accuracy of serum osmolality estimation by different formulas during the administration of hyperosmolar agent.

**Methods:**

A prospective, randomized, double-blinded, controlled trial was conducted in a 30-bed neurosurgical intensive care unit at a university hospital. Thirty-five adult patients requiring the use of hyperosmolar agents for prevention or treatment of brain edema after elective craniotomy were enrolled, and randomly assigned 1:1 to receive 125 mL of either 20 % mannitol (mannitol group) or 3.1 % sodium chloride solution (hypertonic saline group) in 15 min. Serum osmolality, serum sodium and potassium concentration, blood urea nitrogen and blood glucose concentration were measured during the study period. The primary outcome was the agreement of measured and estimated serum osmolality during the infusion of the two experimental agents. We used Bland and Altman’s limits of agreement analysis to clarify the accuracy of estimated serum osmolality. Bias and upper and lower limits of agreement of bias were calculated.

**Results:**

For each formula, the bias was statistically lower in hypertonic saline group than mannitol group (*p* < 0.001). Within group comparison showed that the lowest bias (6.0 [limits of agreement: −18.2 to 30.2] and 0.8 [−12.9 to 14.5] mOsml/kg in mannitol group and hypertonic saline group, respectively) was derived from the formula ‘2 × ([serum sodium] + [serum potassium]) + [blood urea nitrogen] + [blood glucose]’.

**Conclusions:**

Compared to mannitol, a better agreement between measured and estimated serum osmolality was found during the infusion of hypertonic saline. This result indicates that, if hypertonic saline is chosen to prevent or treat brain edema, calculated serum osmolality can be used as a reliable surrogate for osmolality measurement.

**Trial registration:**

ClinicalTrials.gov identifier: NCT02037815

## Background

Brain edema and elevated intracranial pressure (ICP) are potentially devastating complications following various types of intracranial operations [1–3], and appropriate treatments improve cerebral perfusion and reduce damage by local compression of brain tissue [4, 5]. Hyperosmolar agents have been used to ameliorate brain edema and intracranial hypertension during and after craniotomy, where mannitol and hypertonic saline (HS) are the two most extensively studied and most frequently used in the clinical practice [6–12]. Although recent meta-analyses suggested that HS might be more effective than mannitol in controlling intracranial hypertension, no significant differences have been found in neurological outcome and side effects between the two agents [13–16].

The primary mechanism of hyperosmolar agents to control brain edema is based on the increased osmotic gradient across blood–brain barrier during drug infusion, and this helps in the removal of water from brain tissue to the intravascular space [17]. Clinical studies showed that an osmotic gradient between blood and brain of just above 10 mOsmol/kg was effective in reducing ICP [18]. In clinical practice, serum osmolality can be used as a surrogate measure of the effect of hyperosmolar agents, with either mannitol or HS. The initial target of serum osmolality is often set slightly above the upper limit of normal range [10]. Acute renal failure might develop when serum osmolality exceeds 320 mOsmol/kg during mannitol infusion [19]. Therefore, measurement of serum osmolality during hyperosmolar agent infusion is of clinical importance to determine clinical efficacy, adjust dosage and avoid side effects.

Serum osmolality is often measured in laboratory by cryoscopic technique as the reference method [20]. However, in clinical setting, routine measurement of serum osmolality is not always feasible at bedside, either in intensive care unit (ICU) or in the neurosurgical ward. In this situation, clinicians usually estimate serum osmolality by using formulas derived from serum osmoles that can be measured by bedside blood gas analysis or routine laboratory chemical analysis, such as serum sodium, potassium, urea, and glucose [21]. However, several studies have shown that during mannitol infusion, calculated serum osmolality may lead to a systematic bias compared to direct measurement [22–24]. This poor agreement of measured and calculated osmolality during mannitol infusion might be due to the osmolal gap, which is the difference between the two values. Up to now, few studies have been carried out to determine the accuracy of serum osmolality estimation during HS infusion [23]. In the present study, mannitol or HS was used in patients after elective craniotomy, and serum osmolality was measured during drug infusion. Four most cited formulas were chosen to estimate the serum osmolality [21]. Parameters in these formulas are easily collected in clinical practice. The aim was to determine the accuracy of serum osmolality estimation during the application of hyperosmolar agents. We hypothesized that the agreement of measured and calculated serum osmolality during the infusion of HS would be better than mannitol.

## Methods

### IRB/Consent

The trial complied with the latest Declaration of Helsinki. The study protocol was approved by the Institutional Review Board (IRB) of Beijing Tiantan Hospital, Capital Medical University (KY-2013-002-003). Patients’ conscious state was impaired after surgery; therefore, we obtained written consent from patients’ relatives.

### Clinical Trial Registration

The study was registered on January 16, 2014 at the ClinicalTrials.org (NCT02037815). The study protocol was published in April, 2014 [25]. There were two major changes in data analysis. First, the primary outcome was changed from the correlation of measured and estimated serum osmolality to the agreement of these two parameters. Second, the limits of agreement analysis was changed to Bland-Altman method for repeated data.

### Study design, setting and participants

We conducted a prospective, randomized, double-blinded, controlled, parallel-group trial in the neurosurgical ICU (30-bed), Beijing Tiantan Hospital (1100-bed), Capital Medical University, Beijing, China, from January to May 2014.

All patients after elective intracranial surgery admitted to our ICU were screened daily for study eligibility.

Inclusion criteria were as follows:

- Age between 18 and 65 years;
- Within 24 h after operation;
- Hyperosmolar agents were required for the prevention or treatment of post-operative brain edema.

Exclusion criteria were:

- History of diabetes;
- History of alcohol abuse;
- Herniation of brain;
- Unstable hemodynamic condition: systolic blood pressure (BP) less than 90 mmHg or need for continuous infusion of vasopressor;
- Presence of oliguric renal failure;
- Serum sodium concentration below 130 mmol/L or above 155 mmol/L;
- Enrolled in another trial.

Patients were enrolled only once unless they were discharged from the hospital and were readmitted beyond 180 days of the first enrollment.

After randomization (computer generated random digits table in sealed and numbered envelopes), enrolled patients were assigned 1:1 to receive 125 mL of either 20 % mannitol (M group) or 3.1 % sodium chloride solution (HS group). A pharmacist filled 125 mL 20 % mannitol or 3.1 % sodium chloride solution into a sterile 250 mL glass bottle. Patients and all study personnel except the investigative pharmacist were blind to treatment assignment. These concentrations and doses of hyperosmolar agents were chosen according to our standard clinical practice for the prevention and treatment of post-operative brain edema in patients after craniotomy. Indications included intracranial ICP above 25 mmHg, brain edema shown by CT, or poor neurological status considered due to brain edema and bulging of brain during operation. In our institute, the ICP monitoring was performed in patients with external ventricular drainage (Medtronic Neurosurgery, Goleta, CA, USA). Hyperosmolar agents were initiated in some cases because of clinical decline or elevated ICP, but in many others they were instituted based on CT results or empirically.

### Data collection and trial intervention

At study entry, data on demography, body mass index, history of illness, diagnoses of the patients, duration of operation, and type and amount of hyperosmolar agents used during 24 h before the study drug infusion were recorded. Acute Physiology and Chronic Health Evaluation II score (APACHE II) was calculated. Venous blood sample (3 mL) was obtained and concentrations of serum glucose, triglyceride, cholesterase, albumin, globulins, total serum protein and blood urea nitrogen (BUN) were measured by standard central laboratory device. The reasons for the use of experimental hyperosmolar agents were also documented.

After enrollment of the patient, 125 ml of either 20 % mannitol (M group) or 3.1 % sodium chloride solution (HS group) was infused via central venous line in 15 min by using an infusion pump. Type of fluid intake, cumulative fluid intake, urine output and fluid balance (intake minus output) were documented immediately before the infusion of study agents (T0), 15 min (T15min), 30 min (T30min), 60 min (T60min), 120 min (T120min), 240 min (T240min) and 360 min (T360min) after the start of experimental agents’ infusion. At the same time points, 3 mL arterial blood sample and 10 mL urine sample were collected. Serum and urine osmolality were measured by means of freezing point depression [20]. Blood values of sodium, potassium, and glucosewere measured using an ICU bedside blood gas analyzer. Urine specific gravity and concentration of sodium were also measured by standard central laboratory devices.

### Calculation of serum osmolality and osmolal gap

We used four formulas to estimate serum osmolality [21]

2 × [Na^+^](Formula 1)

2 × ([Na^+^] + [K^+^]) + BG + BUN (Formula 2)

2 × [Na^+^] + 0.9 × BG + 0.93 × BUN × 0.5 (Formula 3)

1.9 × ([Na^+^] + [K^+^]) + BG + BUN × 0.5 + 5 (Formula 4)

[Na^+^], serum sodium concentration (mmol/L); [K^+^], serum potassium concentration (mmol/L); BG, blood glucose concentration (mmol/L); BUN, blood urea nitrogen (mmol/L).

Osmolal gap was calculated as the difference between the measured values and each of the estimated values by different formulas shown above.

### Outcome measures

The primary outcome was the agreement of measured and estimated serum osmolality (osmolal gap) during the infusion of hyperosmolar agent. The differences between the measured and each of the calculated values from different formulas were compared. Other outcome measures included changes in the following values during the infusion of experimental agents: serum and urine osmolality, serum and urine sodium concentration, urine specific gravity and fluid balance variables.

### Statistical analysis

Previous investigation showed that by using the formula 2 listed above, the peak serum osmolal gap during mannitol infusion was 25 mOsmol/kg in patients with brain injury [22]. We hypothesized that the serum peak osmolal gap would decrease to 15 mOsmol/kg during the infusion of 3.1 % sodium chloride solution. Using the Power and Sample Size Calculation program, we needed to enroll 15 patients in each group to be able to reject the null hypothesis that the mean peak serum osmolal gap of the two experimental groups was equal with a probability (power) of 0.8. The Type I error probability with testing this null hypothesis was 0.05.

All analyses were according to the intention-to-treat principle, that was, all randomized patients were analysed in the groups to which they had been originally allocated and were blinded to treatment assignment.

Categorical variables were presented as numbers and percentages and analyzed by the *χ*^2^ test. Continuous variables were checked for normal distribution and presented as mean and SD or median and IQR as appropriate. Comparison of continuous variables was performed by using Student *t* test for normally distributed variables and the Mann–Whitney *U* test for non-normally distributed variables.

We used Bland and Altman’s [26] limits of agreement analysis to clarify the accuracy of estimated serum osmolality calculated by each of the four formulas listed above. Bias was defined as the mean of the difference between measured and calculated values (measured minus calculated). SD of the mean bias was calculated according to the agreement between methods of measurement with multiple observations per individual [27]. Upper and lower limits of agreement were defined as bias ± 1.96 SD of the mean bias.

We used repeated measures of analysis of variance for comparing serum osmolal gap, serum and urine osmolality, serum and urine sodium concentration, urine specific gravity and fluid balance variables across different time points (T0 to T360min) between the two groups (M and HS groups).

All tests of significance were at 0.05. Analyses were conducted using SPSS V.17.0.

## Results

**Fig. 1:**
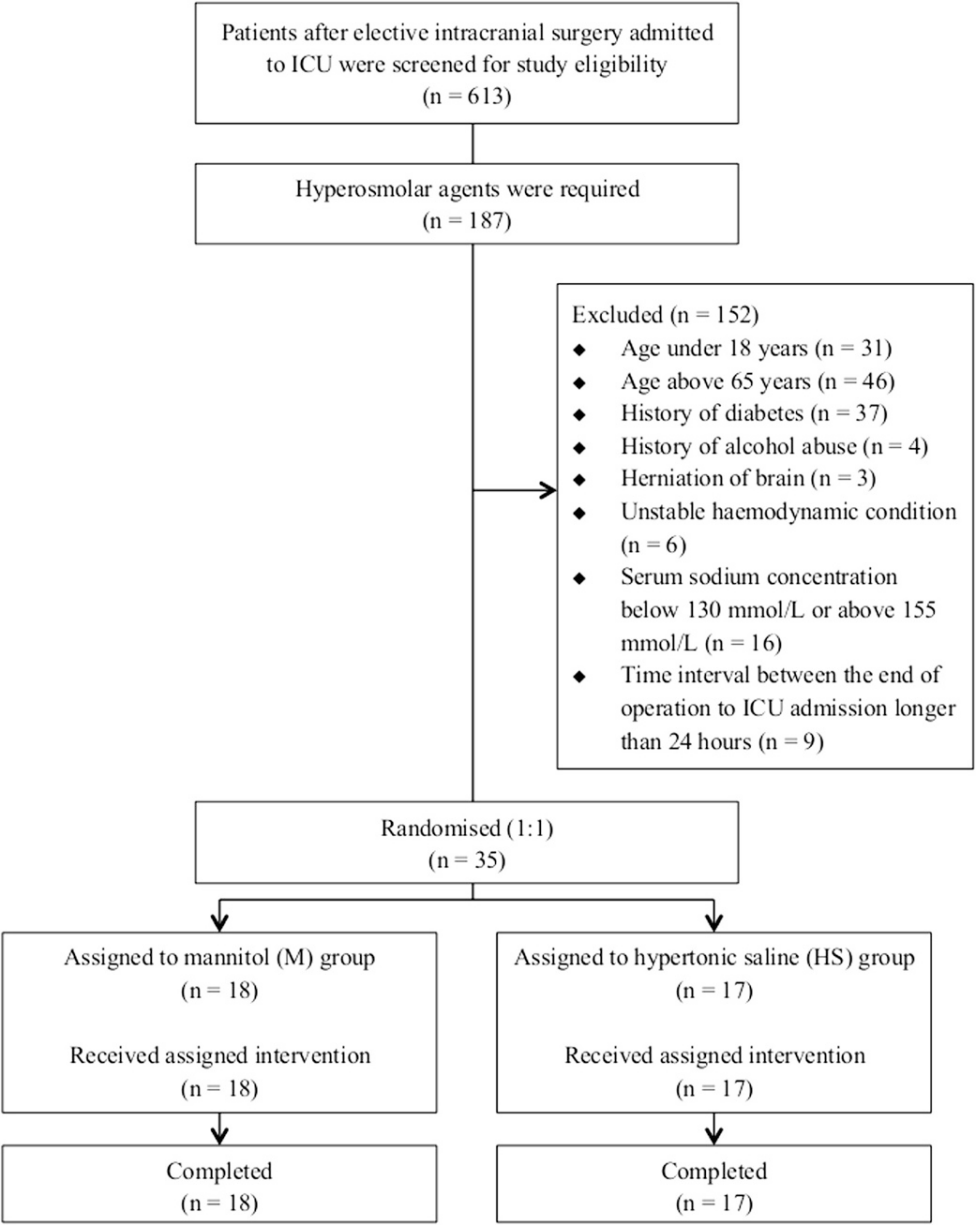
The flow chart of patient participation

Between January and May 2014, 613 patients after elective intracranial surgery were admitted to the ICU and screened for study eligibility. Hyperosmolar agents were required in 187 patients for the prevention or treatment of post-operative brain edema. Among these patients, 35 were enrolled and randomly assigned to M group or HS group. Figure 1 shows the flow chart of patient participation. All patients completed the study intervention and data analysis.

The enrolled patients comprised 18 males (51 %) and 17 females (49 %) with mean (±SD) age of 45 (±12) years. All patients’ diagnoses were brain tumor. Baseline characteristics are shown in Table 1.

**Table 1:** Baseline characteristics of the patients according to randomization groups

Characteristics	M group (*n* = 18)	HS group (*n* = 17)
Age, years	44 ± 14	46 ± 10
Male	9 (50.0 %)	9 (52.9 %)
Body mass index	23.3 ± 4.7	24.5 ± 2.5
Operation time, h	5.1 ± 1.3	4.0 ± 1.0
Use of mannitol before the study		
Incidence	4 (22 %)	5 (29 %)
Time of use before the study, h	10.3 ± 3.3	12.6 ± 3.3
Serum biochemistry		
Sodium, mmol/L	137 ± 2	138 ± 3
Potassium, mmol/L	3.5 ± 0.3	3.5 ± 0.4
Glucose, mmol/L	6.66 ± 1.80	6.83 ± 1.64
Triglyceride, mmol/L	1.57 ± 0.27	1.64 ± 0.21
Cholesterase, mmol/L	4.21 ± 0.75	4.32 ± 1.02
Albumin, g/L	37.6 ± 3.4	36.8 ± 4.5
Globulins, g/L	24.8 ± 3.9	23.7 ± 3.2
Total protein, g/L	62.4 ± 6.6	60.5 ± 7.1
Blood urea nitrogen, mmol/L	3.9 ± 1.8	4.0 ± 1.2
APACHE II	15 ± 2	14 ± 3
Reason for use of hyperosmolar agents		
Treatment	5 (28 %)	5 (29 %)
Prevention	13 (72 %)	12 (71 %)

Data are presented as mean ± SD or number (%). APACHE II: Acute Physiology and Chronic Health Evaluation II score

Serum osmolality were measured 7 times in each patient, from immediately before (T0) to 360 min (T360min) after the start of experimental agentsinfusion. At the same time points, blood concentrations of sodium, potassium, and glucose were also measured. Blood concentration of BUN was only measured at T0. Four formulas were used to calculate serum osmolality. So, 245 paired data sets (126 in M group and 119 in HS group) were obtained, which comprised measured and four estimated serum osmolality. Table 2 shows the results of Bland and Altman’s limits of agreement analysis. For each formula, bias was statistically lower in HS group than in M group (*p* < 0.001). Within group comparison showed that, either in M group or in HS group, there was significant difference in bias across any of the four formulas (*p* < 0.001). The lowest bias with narrowest limits of agreement between the measured and estimated serum osmolality was derived from the formula 2, with 6.0 (−18.2 to 30.2) and 0.8 (−12.9 to 14.5) mOsmol/kg in M group and HS group, respectively (Table 2).

**Table 2:** Bland and Altman’s limits of agreement analysis between measured and calculated serum osmolality (mOsmol/kg) by the four formulas

Formulas	M group (126 pairs)		HS group (119 pairs)	
	Bias	Limits of agreement	Bias	Limits of agreement
1	25.0	2.6 to 47.5	19.5	5.1 to 33.8
2	6.0	−18.2 to 30.2	0.8	−12.9 to 14.5
3	16.3	−7.5 to 40.2	11.3	−2.1 to 24.8
4	17.3	−6.2 to 40.7	12.6	−0.6 to 25.7

Data are presented as bias and lower to upper limits of agreement. The differences between measured and calculated serum osmolality were calculated in each data set. Bias was defined as the mean of difference between the measured and calculated values. SD of the mean bias was calculated according to the agreement between methods of measurement with multiple observations per individual. Upper and lower limits of agreement were defined as bias ± 1.96 SD

Patients in M group or HS group received 125 ml of either 20 % mannitol or 3.1 % sodium chloride solution, respectively

For each formula, the bias in HS group was statistically lower than in M group (*p* < 0.001). Within group comparison showed that, either in M group or in HS group, there was significant difference in bias between any of the two formulas (*p* < 0.001)

Table 3 shows variables of osmolality, electrolytes, and fluid balance during the infusion of experimental agents. After the infusion of hyperosmolar agents, serum osmolality increased statistically to the peak value (301.6 ± 10.5 [*p* < 0.001] and 299.6 ± 7.3 [*p* < 0.001] mOsmol/kg in M group and HS group, respectively) at T15min in both groups, and no significant differences were found between the two groups at any time point (*p* = 0.515). In M group, serum osmolality at T15min and T30min were statistically higher than values at baseline and other time points (*p* < 0.05), whereas values at T240min and T360min decreased statistically below the baseline value (*p* < 0.05). The change of serum osmolality in HS group exhibited the same tendency as in M group. However, in HS group, serum osmolality at T240min (*p* = 0.810) and T360min (*p* = 0.076) were not statistically different from baseline value.

**Table 3:** Variables of osmolality, electrolytes, and fluid balance during the infusion of experimental agents

Variables	Time	M group	HS group	*p* value
Serum osmolality, mOsmol/kg	T_0_	296.3 ± 11.2^bcfg^	295.7 ± 7.4^bcd^	0.847
	T_15min_	301.6 ± 10.5^acdefg^	299.6 ± 7.3^acdefg^	0.515
	T_30min_	298.9 ± 10.1^abdefg^	298.4 ± 7.4^abefg^	0.875
	T_60min_	297.0 ± 8.3^bcfg^	297.8 ± 7.0^abfg^	0.770
	T_120min_	296.7 ± 9.7^bcfg^	296.6 ± 6.6^bcg^	0.963
	T_240min_	294.9 ± 10.3^abcdeg^	295.9 ± 6.5^bcdg^	0.751
	T_360min_	292.4 ± 9.1^abcdef^	294.1 ± 6.6^bcdef^	0.527
Serum osmolal gap*, mOsmol/kg	T_0_	3.6 ± 12.1^bcgf^	2.2 ± 6.9^b^	0.684
	T_15min_	16.4 ± 11.4^acdefg^	−0.9 ± 6.2^acd^	<0.001
	T_30min_	10.8 ± 12.3^abdefg^	2.0 ± 6.9^b^	0.014
	T_60min_	5.3 ± 8.9^bcfg^	1.3 ± 6.8^b^	0.142
	T_120min_	3.7 ± 11.1^bcg^	0.8 ± 7.2	0.368
	T_240min_	2.4 ± 12.1^bcd^	−0.1 ± 8.1	0.470
	T_360min_	−0.1 ± 10.1^abcde^	0.2 ± 6.5	0.905
Serum sodium, mmol/L	T_0_	137.0 ± 2.1^bc^	137.3 ± 2.8^bcd^	0.723
	T_15min_	133.6 ± 2.6^acdefg^	141.4 ± 3.1^acdefg^	<0.001
	T_30min_	134.9 ± 3.1^abdef^	139.2 ± 3.5^abg^	0.001
	T_60min_	136.6 ± 2.8^bc^	139.1 ± 3.2^abg^	0.018
	T_120min_	136.8 ± 3.4^bc^	138.5 ± 3.7^b^	0.164
	T_240min_	136.4 ± 3.2^bc^	138.1 ± 3.3^b^	0.139
	T_360min_	136.1 ± 3.5^b^	137.5 ± 2.9^bcd^	0.202
Urine osmolality, mOsmol/kg	T_0_	825.8 ± 92.1^bcde^	824.2 ± 181.3^bcdef^	0.973
	T_15min_	659.0 ± 123.0^aefg^	727.4 ± 181.1^a^	0.198
	T_30min_	638.1 ± 80.1^adefg^	692.6 ± 186.2^adfg^	0.264
	T_60min_	677.7 ± 79.0^acefg^	731.1 ± 169.2^ac^	0.236
	T_120min_	727.3 ± 84.9^abcdfg^	719.3 ± 182.2^a^	0.868
	T_240min_	788.5 ± 92.0^bcde^	738.7 ± 209.3^ac^	0.354
	T_360min_	808.9 ± 162.9^bcde^	759.8 ± 224.3^c^	0.461
Urine sodium, mmol/L	T_0_	199.2 ± 44.2^bcdefg^	218.8 ± 63.3^f^	0.294
	T_15min_	115.4 ± 30.6^af^	210.5 ± 59.8^f^	<0.001
	T_30min_	106.7 ± 22.1^adfg^	214.7 ± 44.4^ef^	<0.001
	T_60min_	116.6 ± 30.7^acf^	214.6 ± 47.8^ef^	<0.001
	T_120min_	116.8 ± 36.5^af^	199.1 ± 50.1^cd^	<0.001
	T_240min_	140.9 ± 33.5^abcde^	184.7 ± 51.7^abcd^	0.005
	T_360min_	137.4 ± 53.0^ac^	195.1 ± 56.3^d^	0.004
Urine SG, kg/m^3^	T_0_	1.030 ± 0.007	1.028 ± 0.007^bcdefg^	0.421
	T_15min_	1.029 ± 0.006^e^	1.023 ± 0.007^a^	0.007
	T_30min_	1.029 ± 0.006^e^	1.021 ± 0.007^adef^	<0.001
	T_60min_	1.031 ± 0.006	1.022 ± 0.006^ac^	<0.001
	T_120min_	1.033 ± 0.006^bc^	1.022 ± 0.006^ac^	<0.001
	T_240min_	1.032 ± 0.006	1.023 ± 0.007^ac^	<0.001
	T_360min_	1.030 ± 0.009	1.023 ± 0.007^a^	0.008
Cumulative fluid intake, mL	T_0_	0	0	/
	T_15min_	132 ± 16	131 ± 18	0.973
	T_30min_	179 ± 23	187 ± 30	0.419
	T_60min_	280 ± 84	257 ± 51	0.344
	T_120min_	423 ± 102	381 ± 93	0.218
	T_240min_	617 ± 144	647 ± 139	0.536
	T_360min_	813 ± 186	855 ± 219	0.537
Cumulative urine output, mL	T_0_	0	0	/
	T_15min_	76 ± 45	49 ± 22	0.035
	T_30min_	167 ± 66	94 ± 37	<0.001
	T_60min_	300 ± 108	171 ± 71	<0.001
	T_120min_	449 ± 155	303 ± 121	0.004
	T_240min_	662 ± 183	537 ± 189	0.050
	T_360min_	835 ± 221	775 ± 253	0.455
Cumulative fluid balance^†^, mL	T_0_	0	0	/
	T_15min_	56 ± 47	83 ± 28	0.051
	T_30min_	12 ± 69	93 ± 49	<0.001
	T_60min_	−20 ± 139	86 ± 84	0.010
	T_120min_	−26 ± 179	79 ± 131	0.058
	T_240min_	−45 ± 233	110 ± 194	0.042
	T_360min_	−23 ± 267	81 ± 255	0.250

Data are presented as mean ± SD

*Urine SG* urine specific gravity

*: Calculated osmolality was derived from formula 2

^†^: Fluid balance = intake-output

*P* Values represent between group comparisons. Within group comparisons are labeled as: ^a^statistically different compared to T_0_; ^b^ to T_15min_; ^c^ to T_30min_; ^d^ to T_60min_; ^e^ to T_120min_; ^f^ to T_240min_; ^g^ to T_360min_

**Fig. 2:**
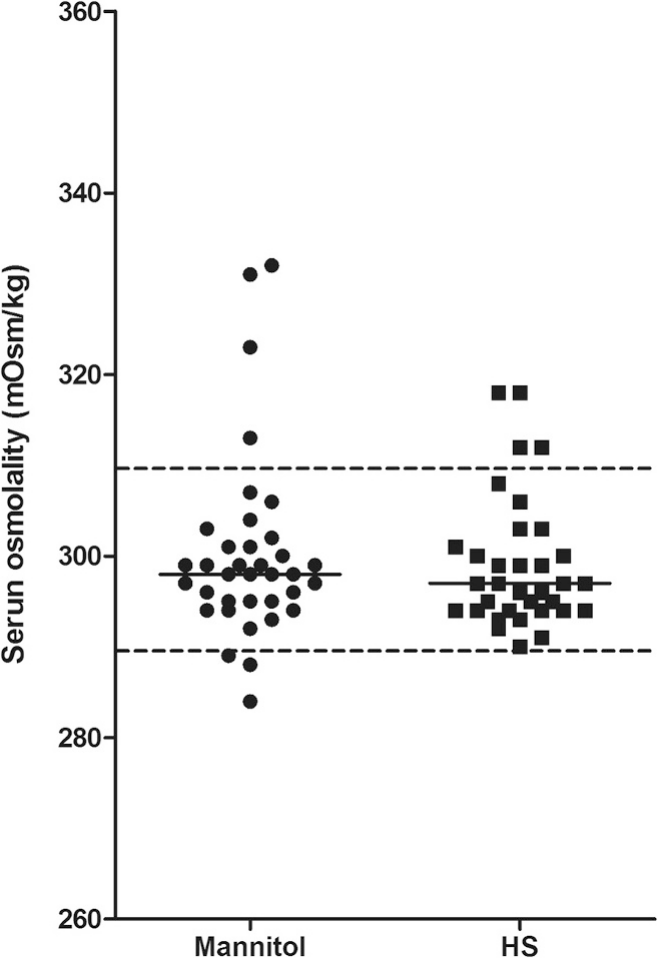
Distribution of serum osmolality at T15min to T30min

Since serum osmolality was higher at T15min and T30min in both M group and HS group, we combined the data at this two time points to determine whether desired serum osmolality was achieved. In M group, 4 (11.1 %) measurements were greater than 310 mOsmol/kg, 3 (8.3 %) measurements were lower than 290 mOsmol/kg, while 27 (80.6 %) measurements were between 290 and 310 mOsmol/kg. In HS group, 4 (11.8 %) measurements were greater than 310 mOsmol/kg while other measurements were between 290 and 310 mOsmol/kg (Fig. 2).

Because the lowest bias between measured and estimated serum osmolality was derived from the formula 2 (Table 2), serum osmolal gap was calculated according to this formula. In M group, after the infusion of mannitol, osmolal gap increased statistically at T15min (16.4 ± 11.4 mOsmol/kg, *p* < 0.001) and T30min (10.8 ± 12.3 mOsmol/kg, *p* < 0.001), and decreased to the baseline level at time points of T60min to T240min, then further decreased statistically below the baseline value at T360min (*p* = 0.002, Table 3). In HS group, the osmolal gap at T15min (−0.9 ± 6.2 mOsmol/kg, *p* = 0.029) was slightly but significantly lower than the baseline value (Table 3). No significant differences were found among values at baseline and at the time points from T30min to T360min (*p* > 0.05). Between groups comparison showed statistically higher osmolal gap at T15min (*p* < 0.001) and T30min (*p* = 0.014) in M group than HS group, but no significant differences existed at time points from T60min to T360min (*p* > 0.05).

After the infusion of experimental agents, tendency of change in serum sodium differed statistically between the two groups (Table 3). In M group, after mannitol infusion, serum sodium decreased statistically from 137.0 ± 2.1 mmol/L at baseline to 133.6 ± 2.6 mmol/L at T15min (*p* < 0.001) and 134.9 ± 3.1 mmol/L at T30min (*p* = 0.001). In HS group, serum sodium increased statistically from 137.3 ± 2.8 mmol/L at baseline to 141.4 ± 3.1 mmol/L at T15min (*p* < 0.001), and maintained statistically greater than baseline at T30min (*p* = 0.002) and T60min (*p* = 0.015).

The tendency of change in urine osmolality did not differ between the two groups (Table 3). After the infusion of study agents, urine osmolality decreased statistically at time points from T15min to T120min (*p* < 0.05), and then recovered to baseline level. For urine sodium, significant differences were found between the two groups at all time points after the infusion of study agents (*p* < 0.05). After the infusion of mannitol, urine sodium decreased statistically (*p* < 0.001). However, in HS group, urine sodium did not change statistically (*p* > 0.05, only slightly decreased at T240min [*p* = 0.007]). Significant difference was also found in urine specific gravity after study agents’ infusion between the two groups (*p* < 0.05). Urine specific gravity in M group did not change statistically during the study (*p* > 0.05), but in HS group, this parameter decreased statistically after infusion and remained in the level below baseline (*p* < 0.05).

We also collected data of fluid balance during the study period (Table 3). For cumulative fluid intake, there was no significant difference between the two groups (*p* > 0.05). However, cumulative urine output in M group was statistically higher than HS group, from T15min to T240min (662 ± 183 mL in M group and 537 ± 189 mL in HS group, *p* < 0.05). This resulted in the significant differences in cumulative fluid balance between the two groups. Cumulative fluid balance was statistically more negative in M group than HS group at T30min, T60min and T240min (*p* < 0.05).

## Discussion

In this prospective, double-blinded, randomized controlled trial, we determined the agreement of measured and estimated serum osmolality during theoretically equiosmolar load of mannitol and HS, and compared the accuracy of serum osmolality estimation derived from four common used formulas between the two agents. The main findings of our study are that, (1) calculation by using the formula ‘2 × ([Na^+^] + [K^+^]) + BUN + BG’ provides the most accurate estimation of serum osmolality after the infusion of HS; (2) an increase of serum osmolality is observed after the infusion of either 20 % mannitol or 3.1 % HS, and with the latter this is associated with the increase in serum sodium concentration; and (3) compared with HS, mannitol results in an abrupt and significant increase in osmolal gap during the early stage after the infusion.

Hyperosmolar agents are frequently used in prevention and treatment of brain edema during and after craniotomy and monitoring of serum osmolality is essential in this situation [6–12]. A lot of formulas have been published for the application of serum osmolality estimation in general patients population [21]. Among these formulas, several have been evaluated in neurological and neurosurgical patients during the administration of hyperosmolar agents [22–24]. In these studies, different formulas were used and different patients were enrolled (not treated with hyperosmolar agents [24], treated with mannitol [22] or treated with both mannitol and HS [23]). The results showed a suboptimal correlation between measured and calculated serum osmolality during mannitol administration [22, 23]. Although patients receiving mannitol and HS were both enrolled in the study of Vialet et al., results of mannitol and HS were not reported separately [23]. In present study, we chose 4 most cited formulas [21], and confirmed the most accurate formula for the estimation of serum osmolality. Our results indicate that, if HS is chosen to prevent or treat brain edema during post-operative period, calculated serum osmolality can be used as a reliable surrogate for osmolality measurement.

In the present study, the theoretically equiosmolar bolus of either mannitol or HS resulted in an abrupt increase in serum osmolality, reaching the peak value by the end of infusion (Table 3). This is consistent with the results of previous studies [6, 7]. In Rozet et al. study, 5 ml/kg of 20 % mannitol and 3 % HS were administered over 15 min, and both resulted in similar increase in serum osmolality [6]. Francony et al. compared a single infusion of either 231 mL of 20 % mannitol or 100 mL of 7.45 % HS during 20 min of administration [7]. The baseline serum osmolality was not significant different between the two groups, with 296 ± 11 and 292 ± 13 mOsmol/kg in mannitol group and HS group, respectively. Both agents caused 2 % increases in serum osmolality at 30 min after the infusion, also with no difference between the two groups. However, in our study, we found that the magnitude of increase in serum osmolality was higher, although not significantly, after the infusion of 20 % mannitol than 3.1 % HS (Table 3). Calculated osmolality of 20 % mannitol (1098 mosmol/kg) and 3.1 % sodium chloride solution (1054 mosmol/kg) are nearly equal, but measured values are not. By means of freezing point depression, we found that the real osmolality of 20 % mannitol and 3.1 % HS were about 1378 and 972 mOsmol/kg, respectively. According to this result, 4.3 % HS (about 1341 mOsmol/kg) should be used to as the equal-osmolar hyperosmolar agent with 20 % mannitol.

Up to now, only one study investigated osmolal gap during HS infusion. Vialet et al. enrolled 20 patients with traumatic brain injury, who received 2 mL/kg of either 20 % mannitol (*n* = 10) or 7.5 % HS (*n* = 10) in 20 min [23]. By using the formula ‘2 × [Na+] + BUN + BG’, they found an osmolal gap of −2.3 ± 7.2 mOsmol/kg in this population, but unfortunately the respective value in mannitol and HS group was not reported separately. In our study, we calculated the osmolal gap by using the formula ‘2 × ([Na^+^] + [K^+^]) + BUN + BG’, and found a different pattern of change in osmolal gap after the infusion of mannitol and HS (Table 3). Osmolal gap increased statistically at 15 to 30 min after the infusion of mannitol, whereas remained almost unchanged after the infusion of HS (slightly decreased at 15 min). The increasing of serum osmolal gap after mannitol infusion was mainly due to the inverse change of serum sodium concentration after the infusion of the two agents. The dynamic changes of serum sodium during the administration of mannitol and HS in present study were comparable to previous reports [6–9, 28, 29]. The infusion of mannitol attracts water from interstitial space to intravascular space, which results in transient intravascular volume expansion and dilutional hyponatremia.

In the present study, we also determined the influence of hyperosmolar agents on fluid and urine variables (Table 3). Since the cumulative fluid intake was not different between the two groups, higher cumulative urine output after mannitol infusion resulted in a more negative fluid balance. This is in accordance with the effect of osmotic diuresis with mannitol infusion reported in previous studies [6–8, 30, 31]. These results suggest that different strategy should be employed during mannitol and HS therapy. Hypovolemia should be avoided after mannitol infusion, whereas hypervolemia be avoided after HS infusion.

There are some limitations to this study. First, although it was not surprising that we found a lower osmolal gap after the infusion of HS than mannitol, the most reliable formulas for serum osmolality estimation was identified in present study. Second, the doses of mannitol and HS prepared and used in present study were not actually equiosmolar. This is the same problem in other studies for the comparison of equiosmolar hyperosmolar solutions [6–8]. We found that 20 % mannitol (1378 mOsmol/kg) and 4.3 % HS (1341 mOsmol/kg) were measured equiosmolar. Third, we only determined the change of osmolality after single bolus of hyperosmolar agents. In clinical practice, hyperosmolar agents are usually given several times to maintain a hyperosmolar state. Our results cannot be applied to repeated use of hyperosmolar agents. Fourth, we did not investigate the effect of hyperosmolar agents on intracranial pressure and other clinical outcomes. However, this is beyond the scope of our study.

To our knowledge, this is the first prospective, randomizedcontrolled, double-blinded human study comparing the agreement of measured and estimated serum osmolality during the infusion of mannitol and HS. The results indicate that, if HS is chosen to prevent or treat brain edema during post-operative period, calculated serum osmolality can be used as a reliable surrogate for osmolality measurement.

## Conclusions

Compared to mannitol, a better agreement between measured and estimated serum osmolality was found during the infusion of hypertonic saline. This result indicates that, if hypertonic saline is chosen to prevent or treat brain edema, calculated serum osmolality can be used as a reliable surrogate for osmolality measurement.

## Abbreviations

ICP: Intracranial pressure
HS: Hypertonic saline
ICU: Intensive care unit
BP: Blood prssure
M: Mannitol
APACHE II: Acute physiology and chronic health evaluation II
BUN: Blood urea nitrogen
BG: Blood glucose concentration
